# How Repetition Rate Impacts Detection Limits of Ion Mobility Spectrometers with Field-Switching Ion Shutters

**DOI:** 10.1021/acs.analchem.5c01027

**Published:** 2025-04-10

**Authors:** Martin Lippmann, Moritz Hitzemann, Alexander Nitschke, Stefan Zimmermann

**Affiliations:** Department of Sensors and Measurement Technology, Institute of Electrical Engineering and Measurement Technology, Leibniz Universität Hannover, Appelstr. 9A, Hannover 30167, Germany

## Abstract

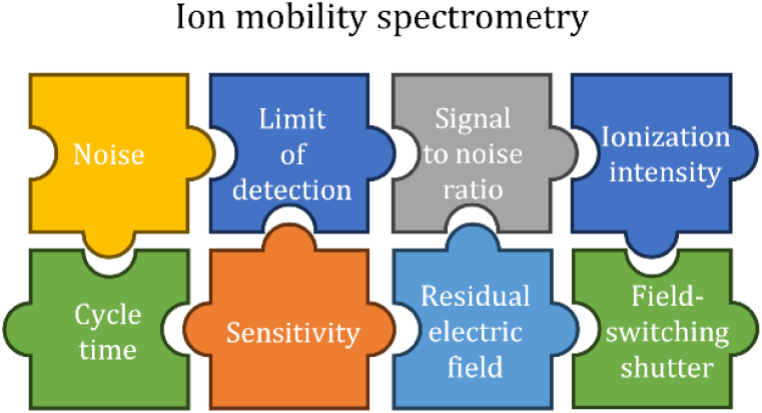

Ion mobility spectrometers are extremely sensitive analytical instruments, consisting of an ionization region and a drift region, usually separated by an ion shutter. Highest sensitivity is reached with field-switching ion shutters, as ions are accumulated in the ionization region while the ion shutter is closed, which defines the reaction time in the ionization region. This study investigates the effect of repetition rate and reaction time on ion formation and detection limits. The results reveal that increasing the ionization source intensity and reaction time increases the signal-to-noise ratio even though less spectra can be averaged in a given overall measuring time. It is shown that the formation of protonated monomers and proton-bound dimers is considerably slower than the formation of reactant ions, highlighting the impact of reaction time on signal-to-noise ratio. At maximum ionization source intensity and optimal reaction times for the protonated monomers and proton-bound dimers of 1-butanol, limits of detection of 1.9 ppt_v_ and 110 ppt_v_ could be reached. For the protonated monomers and proton-bound dimers of 2-butanone, the limits of detection are 1.3 ppt_v_ and 57 ppt_v_. The given limits of detection refer to an averaging time of 1 s. Although the optimal reaction times differ for different protonated monomers and proton-bound dimers, a reaction time of 40 ms was identified as a good compromise. These findings provide valuable insights into how the reaction time, and thus repetition rate and cycle time, impacts the detection limits of ion mobility spectrometers equipped with field-switching ion shutters.

## Introduction

Drift tube ion mobility spectrometers (IMS) using atmospheric pressure chemical ionization (APCI) are extremely sensitive analytical instruments, with limits of detection (LoD) reaching single-digit ppt_v_ values within measurement times of 1 s.^1−5^ Substances are analyzed in an IMS by ionizing the gaseous sample and separating the ions by their specific ion mobility under the influence of an electric field in a drift gas. An IMS usually consists of two distinct regions: the ionization region and the drift region, usually separated by an ion shutter consisting of one or more metallic grids. The ion shutter controls the transfer of short ion packages from the ionization region to the drift region. Within the drift region, ions are accelerated toward the detector by a constant electric field, with their motion periodically slowed by collisions with neutral gas molecules, resulting in a mean drift velocity that is characteristic for each ion depending on its size and mass and giving the characteristic ion mobility.

In the ionization region, ions are generated by various ionization processes and sources, including radioactive ^63^Ni, ^3^H,^6^ corona discharge,^7^ plasma ionization,^8^ electrospray ionization (ESI),^9^ ultraviolet light (UV),^10^ or X-ray radiation.^11^ The choice of the ionization source limits the type of usable ion shutters. A comprehensive review of the shutter principles used in IMS is provided by Chen et al.^12^ The Bradbury–Nielsen (BN) shutter, for example, is a popular ion gate, extensively described in the literature.^13−17^ This ion shutter is used alongside a constant electric field in the ionization region. However, given typical IMS cycle times of around 20 ms and shutter opening times of microseconds, the BN shutter suffers from a low-duty cycle, which reduces ion yield and, thus, sensitivity. The same applies to Tyndall–Powell ion shutters.^12,18^ Consequently, many studies have explored multiplexing techniques to enhance the performance.^19−21^

An alternative ion shutter, first described by McGann et al.,^22^ is the so-called field-switching ion shutter.^6,23^ This ion shutter operates with a field-free ionization region, allowing ions to accumulate during its closed state. Applying an electric field across the ionization region for several microseconds pushes the accumulated ions into the drift region. As such, a field-switching ion shutter requires an ionization source that generates ions effectively in an almost field-free ionization region, such as ^3^H^24^ or X-ray sources.^25^ However, ionization sources that require a larger ionization region or an electrical field, e.g., ^63^Ni, corona discharge, and electrospray ionization, do not fit this concept. Nevertheless, field-switching ion shutters can significantly accumulate ions if an almost field-free ionization region is maintained, outperforming Bradbury–Nielsen and Tyndall–Powell ion shutters in sensitivity.^26,27^ Minimizing the residual electric field inside the ionization region of a field-switching ion shutter, which is generated by the drift field penetration through the openings in the metallic grid, is crucial to fully close the ion shutter and increase the number of accumulated ions. This can be achieved either by metallic grids with small openings or by applying a so-called compensation voltage, generating an electric field that counteracts the penetration of the drift field. However, metallic grids with small openings can reduce ion transmission, so using a compensation voltage is the preferred method for compensating the residual electric field.

Recent advancements in the field-switching ion shutter technology have demonstrated the importance of achieving a nearly field-free ionization region, thereby significantly increasing IMS sensitivity by using an additional grid to shield the ionization region from the drift field as implemented in the so-called extended field-switching ion shutter.^4^ Besides the residual electric field, ion generation in IMS with field-switching ion shutters is influenced by factors such as the ionization source intensity, ion recombination rates, and analyte concentration. As shown by Kirk, the formation of reactant ions can be described by eq 1, where ρ_RIP_ denotes the charge density of the reactant ions, *I*_source_ the ion current of the ionization source, *l*_i_·*A*_det_ the volume of the ionization region, *l*_i_ the length of the ionization region, *A*_det_ the detector cross section, the reduced ion mobility of the reactant ions *K*_0_,_RIP_, the Loschmidt constant *N*_0_, the particle density *N*, *k*_rec_ the recombination rate, and *z·e* the charge of the ion.^28^
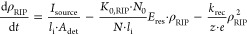
1

As can be seen from this equation, ions are generated by the ionization source, while ion loss can be attributed to the current induced by the residual electric field *E*_res_ and the recombination of positive and negative ions. Since the same quantity of positive and negative ions is accumulated, the loss of ions caused by recombination increases quadratically with the charge density, while the loss due to the residual electric field scales linearly with the charge density and the residual electric field strength.

An additional step is required to ionize the sample molecules, which leads to the analyte ions being analyzed in the IMS. Equation 2 presents the formation of analyte ions based on the formation and loss mechanisms used in eq 1. In this case, analyte ions are formed by interacting with the so-called reactant ions generated in the previous step.
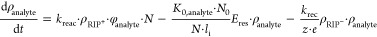
2

In this equation,  describes the ion density of the positive reactant ions,  describes the ion density of the negative reactant ions, ρ_analyte_ describes the ion density of the positive analyte ions, *k*_reac_ the reaction rate coefficient of the analyte with the reactant ions, φ_analyte_ the concentration of the analyte in the sample gas, and *K*_0,analyte_ the reduced ion mobility of the analyte ions. For the analyte ions, the same mechanisms of ion loss as for the reactant ions apply. As both equations demonstrate, ion yield can be improved for a given ionization source by eliminating the residual electric field *E*_res_. Equations 3 and 4 show the stationary solutions for eq 1 and eq 2 without residual electric field and for low concentrations of analytes, where a decrease in reactant ions due to the reaction with analyte ions is negligible.^28^
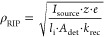
3
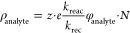
4

Nonetheless, a decisive factor for sensitivity and thus LoD is the time for ions to accumulate in an IMS with field-switching ion shutters since the thermodynamic equilibrium is not reached immediately; therefore, the cycle and reaction time and thus the repetition rate of the IMS have significant influence on the number of accumulated ions. However, the effect of cycle time *t*_c_ on the accumulation of ions has not been investigated experimentally yet, in particular, not in conjunction with the signal-to-noise ratio (SNR) for a given measurement time *t*_m_. While the signal amplitude increases with increasing cycle time before reaching the thermodynamic equilibrium, the noise level also increases as a consequence of a lower number *n* of spectra that can be averaged in the given measurement time *t*_m_, according to eq 5. Thus, the SNR starts decreasing at a certain point, where the increase in noise exceeds the increase in accumulated ions.
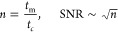
5

Therefore, this work experimentally investigates, for the first time, the effect of the intensity of the ionization source, compensation voltage, and cycle time on ion generation, while considering the effect of cycle time on the noise in order to tune the IMS parameters for optimal LoD.

## Experimental Section

The IMS used for the experiments in this study consists of one of our original drift tube designs^24^ shortened to a drift length of 77 mm and an ionization region based on the ionization source presented in ref (5). In this case, the extended field-switching ion shutter incorporates a stainless-steel pusher electrode and three stainless-steel grids with hexagonal openings and an optical transparency of 80%. The pusher electrode and the two grids adjacent to the ionization region were pulsed in order to control the ion shutter state. The third grid was integrated into the resistive drift voltage divider. This design was chosen as it provides the lowest residual electric field inside the ionization region compared to the original field-switching ion shutter and the extended field-switching ion shutter with two grids as presented in ref (4).

Ions were generated using an orthogonally mounted X-ray source, as already employed in our existing single-polarity X-ray IMS.^5^ The X-ray source is SCXT0829 (Sunje, South Korea). The sample inlet and outlet are constructed of a stainless-steel body featuring a 1 mm hole for the sample gas and a threaded connection for a 1/16″ capillary, secured using Valco nuts and matching ferrules (Vici, Switzerland). All used 1/16″ capillaries made from polyether ether ketone (PEEK) have an inner diameter of 1 mm (211609-10, BGB, Germany). A schematic overview of the IMS with electrical connections is shown in Figure S1.

For this IMS, self-built electronics was used to enhance flexibility and simplify instrument control. The acceleration voltage for the X-ray source was generated by self-built electronics based on a high-voltage DC/DC converter BPN 60 674 (ISEG, Germany). The filament current was regulated by a self-built current controller, supplied by an isolated DC/DC converter REC6-2412SRW/R10/A/X1 (Recom, Germany). For the drift voltage, a high-voltage power supply HVE 35-6500 NEG (FuG, Germany) was used. The generated high voltage was applied to the drift rings and the aperture grid through a resistive drift voltage divider. The pusher electrode and the two switchable grids had separate voltage supplies based on self-built flyback converters, while the compensation voltage applied to the second switchable grid was generated from operational amplifiers. MOSFET half-bridges provided short pulses used for switching between the voltages for injection and ionization state.^29^ For current amplification, a self-built amplifier^30^ with a gain of 5 GΩ and a bandwidth of 25 kHz was used. The signal from the amplifier was digitized at high electric potential with a self-built isolated data acquisition system equipped with a 16-bit analog-to-digital converter.^31^ The operational parameters of the IMS are summarized in Table 1. Although mainly self-built electronics was used in this setup, any commercially available equipment that provides the electric fields and the switching times given in Table 1 may be used instead.

**Table 1:** Operational Parameters of the Ion Mobility Spectrometer

Parameter	Value
Ionization region length	3 mm
Ionization region cross section	12 mm × 10 mm
Drift length	83.5 mm
Drift region diameter	21 mm
Drift field	61 V/mm
Injection pulse width	300 μs
Extension pulse width	500 μs
Injection field	250 V/mm
Pusher grid–extension grid injection field	240 V/mm
Pusher grid–extension grid blocking field	–20 V/mm
Extension grid–injection grid injection field	94 V/mm
Initial ion packet width of reactant ions	30 μs
X-ray acceleration voltage	4.99 kV
Drift gas flow	150 mL/min
Sample gas flow	30 mL/min
Dew point of drift gas and sample gas	183 K (90 ppb_v_ water vapor concentration)
Operating pressure	1025 hPa
Operating temperature of drift tube	298 K
Operating temperature of sample gas inlet	298 K

To explore the influence of various parameters—including the intensity of the ionization source, the compensation voltage, and the cycle time—on ion generation, a series of experiments was conducted. First, the optimal compensation voltage was determined to be 22 V by varying the compensation voltage over a range from 0 to 30 V in increments of 1 V with a fixed filament current of the X-ray ionization source of 400 mA and a fixed cycle time of 30 ms. The subsequent experiments cover full three-dimensional parameter sweeps: compensation voltage, filament current of the X-ray source, and cycle time. Here, the compensation voltage was varied from 2 to 27 V in steps of 5 V, including the optimal compensation voltage of 22 V; the filament current of the X-ray tube was adjusted from 350 to 420 mA in 10 mA increments; and the cycle time was changed from 10 to 100 ms in 2 ms increments. It is noteworthy that the cycle time defines and equals the reaction time. The compensation voltage was varied in these experiments to investigate how the residual electric field strength affects ion formation and to confirm the optimal compensation voltage again. The filament current of the X-ray ionization source was set to a maximum of 420 mA, even though it technically supports up to 460 mA. This decision was made because at 420 mA, the reactant ion peak already approaches the maximum current capability of the current amplifier. Each measurement point of any three-dimensional measurement was repeated three times.

Dried, clean air with a dew point of 183 K was used as drift gas for all experiments. The chemicals 2-butanone (Sigma Product: 360473) and 1-butanol (Sigma product: 19422) were purchased from Sigma-Aldrich, Germany, with a purity >99%. The gas samples were processed using a Vici Dynacalibrator model 150 permeation oven, in which each substance was inserted into self-made permeation tubes and diluted to the desired concentration using a downstream gas dosing device.

The initial measurements were carried out without the presence of any analyte, showing the ion generation of the positive reactant ions (RIP^+^). Subsequently, measurements with nine different concentrations of 1-butanol (48 ppt_v_, 95 ppt_v_, 190 ppt_v_, 379 ppt_v_, 743 ppt_v_, 1520 ppt_v_, 3033 ppt_v_, 6071 ppt_v_, and 12163 ppt_v_) and 13 different concentrations of 2-butanone (39 ppt_v_, 59 ppt_v_, 78 ppt_v_, 117 ppt_v_, 194 ppt_v_, 311 ppt_v_, 467 ppt_v_, 700 ppt_v_, 1088 ppt_v_, 1556 ppt_v_, 2334 ppt_v_, 3889 ppt_v_, and 7794 ppt_v_) were carried out. Concentrations were chosen in a way that some measurements just show the protonated monomers and at least six measurements show both the protonated monomers and the proton-bound dimers. As stated in Table 1, the sample gas containing a constant analyte concentration passes through the reaction region with a constant flow rate of 30 mL/min, so that the volume of the reaction region of 360 μL is exchanged every 720 ms, which also refers to the residence time of analytes in the reaction region. However, as only a fraction of the analyte molecules are ionized^28^ and the residence time of analytes in the reaction region is much longer than the cycle time, the chosen sample gas flow rate does not affect ion formation.

## Results and Discussion

In this work, only the positive polarity was investigated, as the ions in the negative polarity are generated parallel to the positive ions and thus should behave the same way.

In order to demonstrate that the extended field-switching ion shutter reduces the residual electric field inside the ionization region, a series of numerical simulations was conducted using COMSOL Multiphysics. These simulations were then followed by a calculation of the root-mean-square (RMS) value of the residual electric field inside the ionization region for a range of compensation voltages. The results of these calculations are presented in Figure S2. The influence of compensation voltage on RIP^+^ generation was investigated by varying the compensation voltage from 0 to 30 V while maintaining a constant filament current of 400 mA for the X-ray source, corresponding to an X-ray emission current of 7.5 μA and a constant cycle time of 30 ms. Figure S3 shows the RIP^+^ amplitude over the compensation voltage compared to the RMS value of the residual electric field for different compensation voltages obtained from the COMSOL simulation. Up to a compensation voltage of +22 V, the RIP^+^ amplitude increases, while the RIP^+^ amplitude starts to decrease for higher compensation voltages. This aligns well with the simulated residual electric field, which has its minimum voltage at +22 V. Thus, a compensation voltage of +22 V is considered as the optimal operating point.

**Figure 1:**
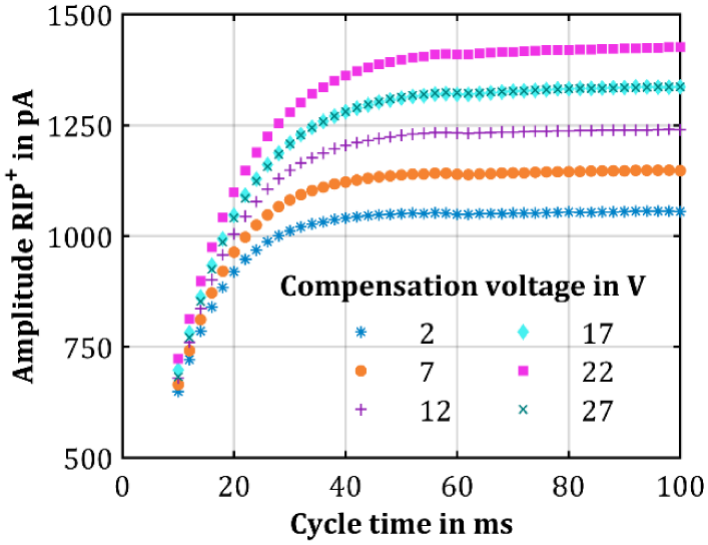
RIP^+^ amplitude of clean, dry air as the sample gas plotted over the cycle time and reaction time, respectively, at different compensation voltages and at a constant filament current of 400 mA.

Figure 1 presents the RIP^+^ amplitude as a function of cycle time and reaction time, respectively, with clean, dry air as the sample gas for six different compensation voltages at an X-ray filament current of 400 mA. These results were obtained by acquiring IMS spectra, with cycle times ranging from 10 to 100 ms, fitting the RIP^+^ with a Gaussian fit, and determining the amplitude from the fitted Gaussian peak. An exemplary IMS spectrum of the RIP^+^ fitted with a Gaussian peak is shown in Figure S4. The data reveal an initially linear increase in amplitude over cycle time, which then levels off for all compensation voltages. However, there is a significant difference in RIP^+^ amplitude for the different compensation voltages. Until the optimal operating point of the compensation voltage, the RIP^+^ amplitude increases with increased compensation voltage for a given cycle time and then decreases again at higher compensation voltages. This behavior is consistent with the results from numerical simulations shown in Figure S3, which indicate that exceeding the optimal compensation voltage will lead to an increasing residual electric field in the ionization region and consequently to a reduced number of ions.

As Figure S5 illustrates, optimizing the compensation voltage to minimize the residual electric field in the ionization region also alters the cycle time required to achieve a steady state in the RIP^+^ amplitude. With a small compensation voltage, the RIP^+^ amplitude levels off after approximately 40 ms. In contrast, the RIP^+^ amplitude levels off after 60 ms with the optimal compensation voltage. However, as shown in Figure 1, for an optimal compensation voltage, the absolute RIP^+^ amplitude at a cycle time of 18 ms is already higher than the value reached with a compensation voltage of 2 V in steady state. In addition, for all cycle times the absolute RIP^+^ amplitude is highest at optimal compensation voltage. This demonstrates how fine-tuning the compensation voltage can maximize the number of ions in the reaction region.

Subsequently, the parameters of the X-ray source were investigated by varying the filament current from 350 to 420 mA with a constantly opened ion shutter and also in regular ionization mode with a cycle time of 30 ms. Figure S6a shows the exponential relationship between filament current and anode current of the X-ray source, while Figure S6b shows the relationship between anode current of the X-ray source and detector current with a constantly opened ion shutter. It seems that the constant detector current depends linearly on the anode current of the X-ray source, whereas in regular ionization mode, the RIP^+^ depends on the square root of the anode current as predicted in eq 3 and as shown in Figure S6c.

**Figure 2:**
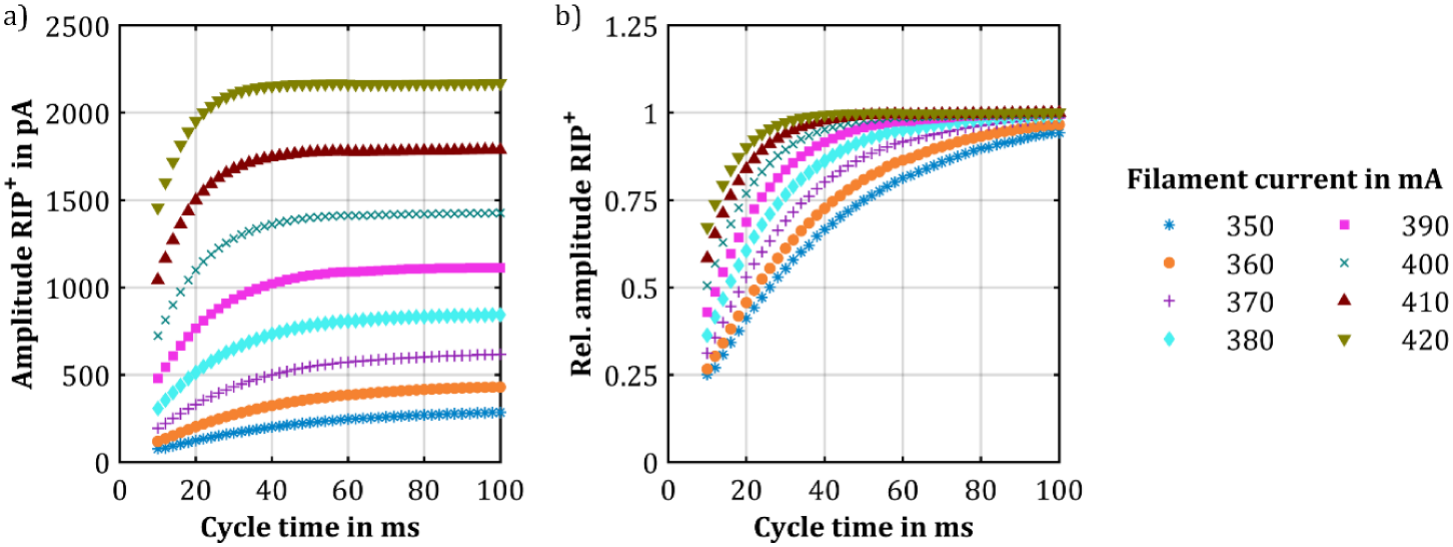
Measurement of (a) the RIP^+^ amplitude and (b) the relative amplitude of the RIP^+^ over the cycle time at optimal compensation voltage but different ionization source intensities. The intensity of the ionization source was varied by adjusting the filament current of the X-ray source.

Next, the influence of the intensity of the X-ray source on the RIP^+^ amplitude was investigated. The measurement confirmed an optimal compensation voltage of 22 V for all intensities of the X-ray source. Therefore, all subsequent results are shown for an optimal compensation voltage of 22 V. Figure 2 shows the RIP^+^ amplitude at the optimal compensation voltage as a function of cycle time for the eight different filament currents of the X-ray ionization source and relative RIP^+^ amplitude (normalized to the RIP^+^ amplitude measured with a very long cycle time of 600 ms) as a function of cycle time. Measurements with cycle times of 200, 400, and 600 ms were made to determine the steady-state value for all settings but were not shown in the figures. The data clearly indicate that an increased X-ray source intensity leads to higher steady-state RIP^+^ amplitudes, which could be expected, and also reduces the time required to reach the steady-state value of the RIP^+^ amplitude. This phenomenon is particularly crucial for IMS applications that require highest repetition rates, as presented by Nitschke et al.,^32^ who coupled an IMS to a hyper-fast GC with short peak widths of just 100 ms. For instance, comparing the filament currents of 420 mA and 370 mA, there is a nearly 4-fold increase in RIP^+^ amplitude at steady state, which expands to a nearly 8-fold increase at a cycle time of 10 ms.

**Figure 3:**
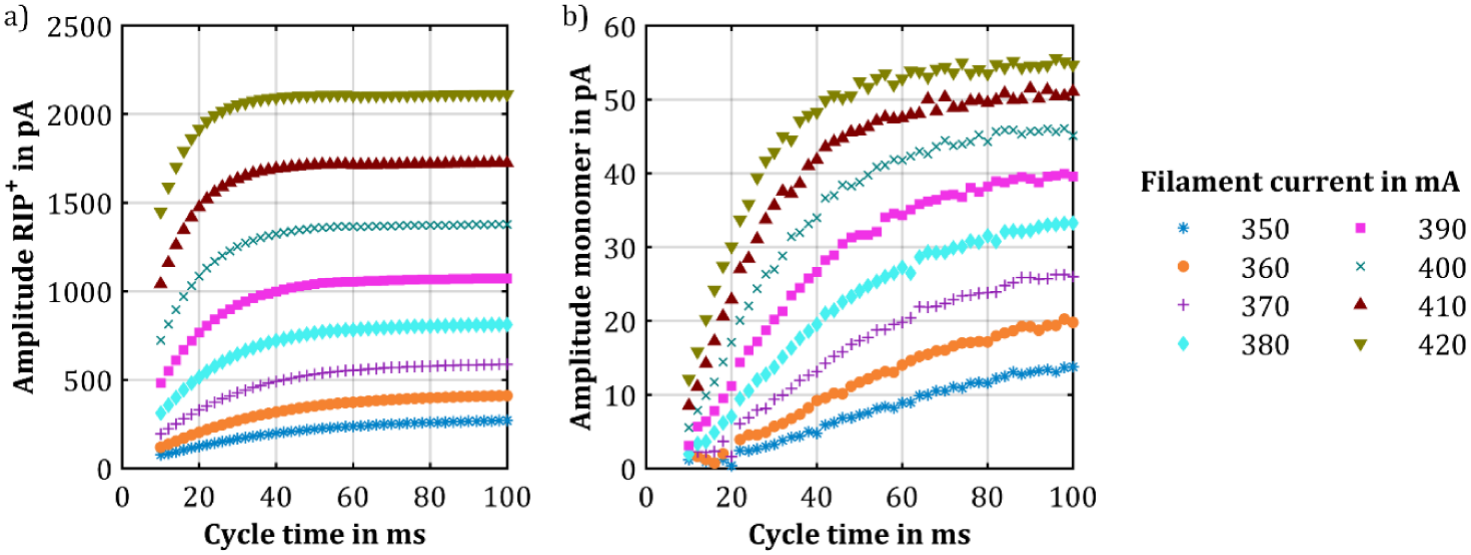
Amplitude of (a) the RIP^+^ and (b) the protonated monomers of 1-butanol plotted over the cycle time at optimal compensation voltage but different ionization source intensities, and 48 ppt_v_ 1-butanol in the sample gas. The intensity of the ionization source was varied by adjusting the filament current of the X-ray source.

After analyzing the generation of the RIP^+^, 48 ppt_v_ of 1-butanol was introduced into the sample gas to evaluate the effect of the intensity of the X-ray source on analyte ion generation at optimal compensation voltage. Figure 3a shows the amplitude of the RIP^+^ as a function of cycle time, while Figure 3b shows the amplitude of the protonated monomers of 1-butanol as a function of cycle time. At this concentration, no proton-bound dimers of 1-butanol could be detected. The results show that at low analyte concentrations, the RIP^+^ amplitude reduces just slightly compared to that observed for pure air. However, compared to the previous measurement, the total charge of positive ions remains nearly the same, as shown in Figure S7. The amplitude of the protonated monomers of 1-butanol increases with cycle time, reaching a steady state at a specific cycle time depending on the used filament current. Similar to the RIP^+^, the amplitude of the protonated monomers increases faster with increased ion source intensity, and the steady state is reached at shorter cycle times. This is also demonstrated in Figure S8a,b, which show the relative amplitude of the RIP^+^ and the protonated monomers of 1-butanol. A comparison of the graphs indicates that the increase in the amplitude of the protonated monomers is significantly slower than that of the RIP^+^. For example, at a filament current of 400 mA, the RIP^+^ reaches a steady state at approximately 50 ms, whereas the steady state for the protonated monomers is reached at around 100 ms. Comparing the amplitude of the protonated monomers at 10 ms with the steady-state value for the same X-ray source intensity reveals a 6- to 7-fold higher amplitude at the steady state.

However, these results do not confirm the behavior suggested by eq 4. In contrast, the amplitudes of the protonated monomers are not independent of the intensity of the ionization source. A definitive explanation for this discrepancy has yet to be identified. One potential explanation could be a residual electric field still present in the ionization region, which might influence the observed results. Alternatively, the analyte concentration might have been too high at this stage to validate eq 4. Furthermore, it is conceivable that eq 4, which is formulated for one specific position inside the ionization region, is not valid for all positions, and thus the measured detector signal no longer represents a valid linear combination of the equation over the entire ionization region. At this point, future investigations are needed to clarify these findings.

**Figure 4:**
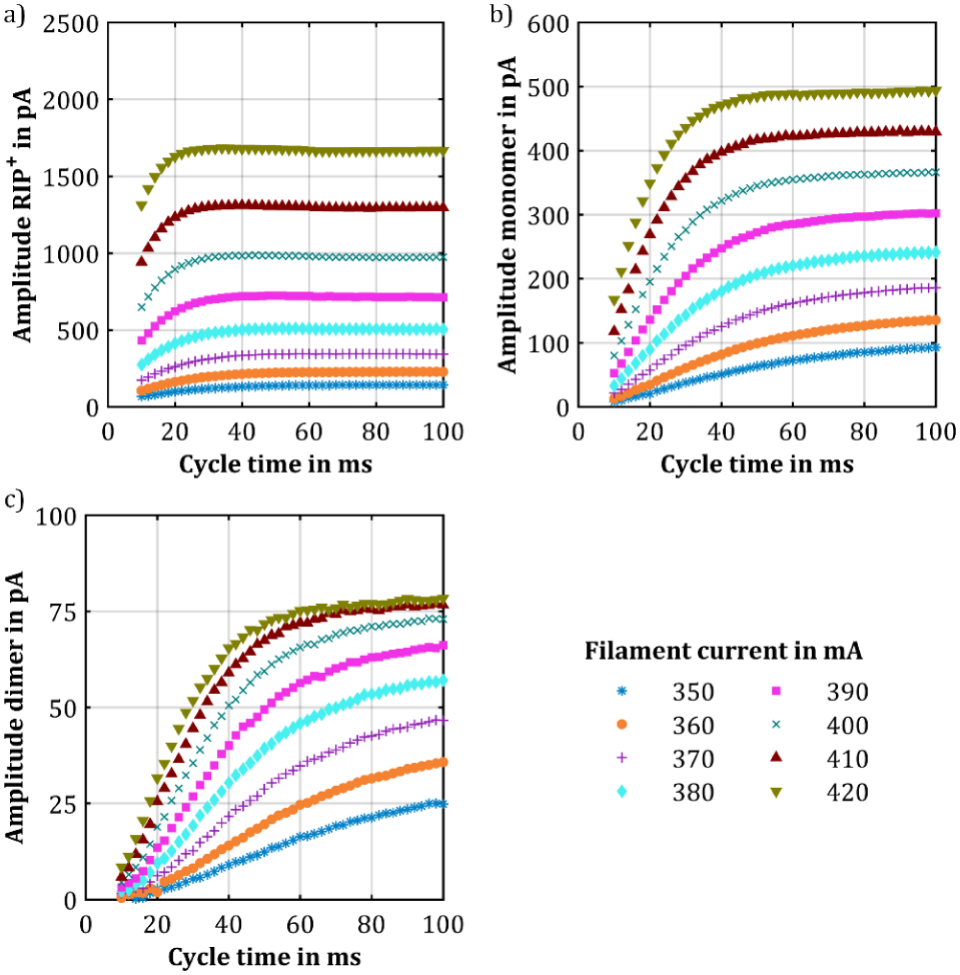
Amplitude of (a) the RIP^+^, (b) the protonated monomers, and (c) the proton-bound dimers of 1-butanol over the cycle time at optimal compensation voltage but different ionization source intensities, and 743 ppt_v_ 1-butanol in the sample gas. The intensity of the ionization source was varied by adjusting the filament current of the X-ray source.

Based on the observation that the protonated monomers form at a slower rate than the RIP^+^, the formation of the proton-bound dimers was also investigated. Therefore, 743 ppt_v_ 1-butanol was introduced to the sample gas. Figure 4a shows the RIP^+^ amplitude as a function of cycle time for different X-ray source intensities, and Figure 4b shows the amplitude of the protonated monomers, while Figure 4c illustrates the amplitude of the proton-bound dimers as a function of cycle time. In this measurement, the steady state of the RIP^+^ amplitude is reached at significantly shorter cycle times than in the measurements without any analyte. Additionally, the absolute RIP^+^ amplitude is markedly reduced. For instance, at a filament current of 400 mA, the RIP^+^ amplitude at steady state is decreased by nearly 30%, whereas the total charge of all ions including the RIP^+^ and the protonated monomers and proton-bound dimers of 1-butanol only decreases by about 5% as shown in Figure S7.

Further, the amplitude of the protonated monomers reaches its steady state at shorter cycle times. However, despite the 15-fold increase in 1-butanol concentration compared to the previous measurement, the amplitude of the protonated monomers, for example, at a filament current of 400 mA, increased by only a factor of approximately 8. This suggests that the concentration range exceeds the linear concentration range of IMS response, where further increasing the analyte concentration does not lead to a proportional increase of analyte ions and thus analyte peak area and amplitude, respectively, as the number of reactant ions available for ionizing further analytes is limited.

Meanwhile, the amplitude of the proton-bound dimers continues to increase over the entire range of cycle times up to 100 ms, without reaching the steady state. The formation of proton-bound dimers is particularly interesting since the ion mobility of proton-bound dimers is less dependent on sample gas humidity.^33,34^ This is important for reducing the likelihood of false-positive or false-negative alarms. Thus, longer cycle times may be beneficial for improving the LoD of proton-bound dimers.

**Figure 5:**
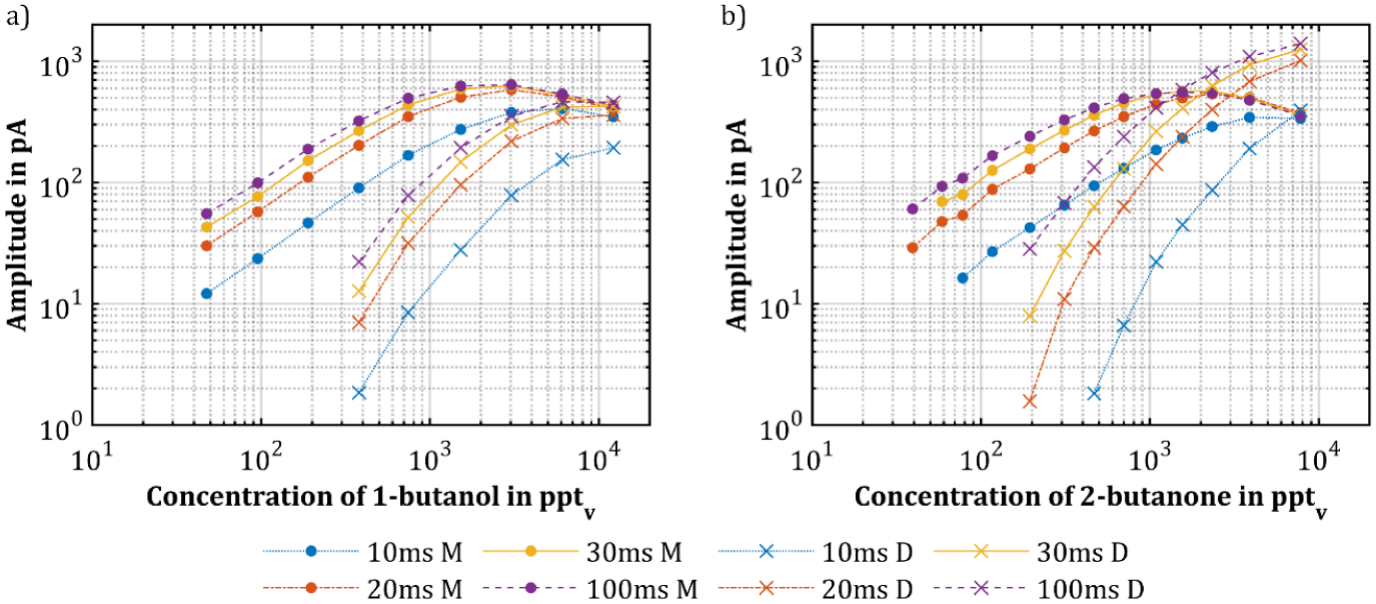
Amplitudes of the protonated monomers (M) and the proton-bound dimers (D) of (a) 1-butanol and (b) 2-butanone plotted over the concentration and at different cycle times.

As demonstrated, the cycle time of IMS with field-switching ion shutters is a critical parameter with respect to sensitivity. To underpin this effect, we analyzed the peak amplitudes measured for different concentrations of 1-butanol and 2-butanone at four cycle times: 10, 20, 30, and 100 ms. Figure 5 shows the amplitudes of the protonated monomers and proton-bound dimers plotted over the concentrations of 1-butanol and 2-butanone, respectively. The results reveal that longer cycle times generally result in higher peak amplitudes for both the protonated monomers and proton-bound dimers. Notably, a cycle time of 10 ms results in significantly lower amplitudes than those obtained for the other three cycle times, despite the time interval between 10 and 20 ms being shorter than that between 20 and 100 ms. This confirms that a substantial amplitude develops even for shorter cycle times. Furthermore, the amplitudes across all cycle times tend to converge when the IMS approaches analyte signal saturation, where the RIP^+^ becomes depleted, particularly evident at higher analyte concentrations. This convergence suggests that the cycle time has less impact on amplitude when leaving the linear IMS response, as available reactant ions limit ion generation.

Finally, the influence of cycle time on the LoD was investigated as the LoD is affected by both the signal amplitude and the noise. In contrast to the peak amplitude reaching a maximum at a certain cycle time, the noise for a given overall measurement time increases with increasing cycle time as fewer averages are possible in a given overall measurement time, as shown in Figure S9. This results in an optimal signal-to-noise ratio at a certain cycle time where the peak amplitude is reasonably high while the noise is still low. Figure S10 exemplarily shows the signal-to-noise ratio of the protonated monomer of 1-butanol depending on the cycle time for a 1-butanol concentration of 48 ppt_v_ and the different filament currents of the X-ray source. Consequently, there is an optimal cycle time for IMS equipped with field-switching ion shutters, where the trade-off between increased amplitude at elongated cycle times and decreased noise due to more averages at shorter cycle times results in optimal LoD. However, given the differing rates of ion formation for protonated monomers and proton-bound dimers, the optimal cycle time depends on the peak considered for detection and the substance under investigation.

**Figure 6:**
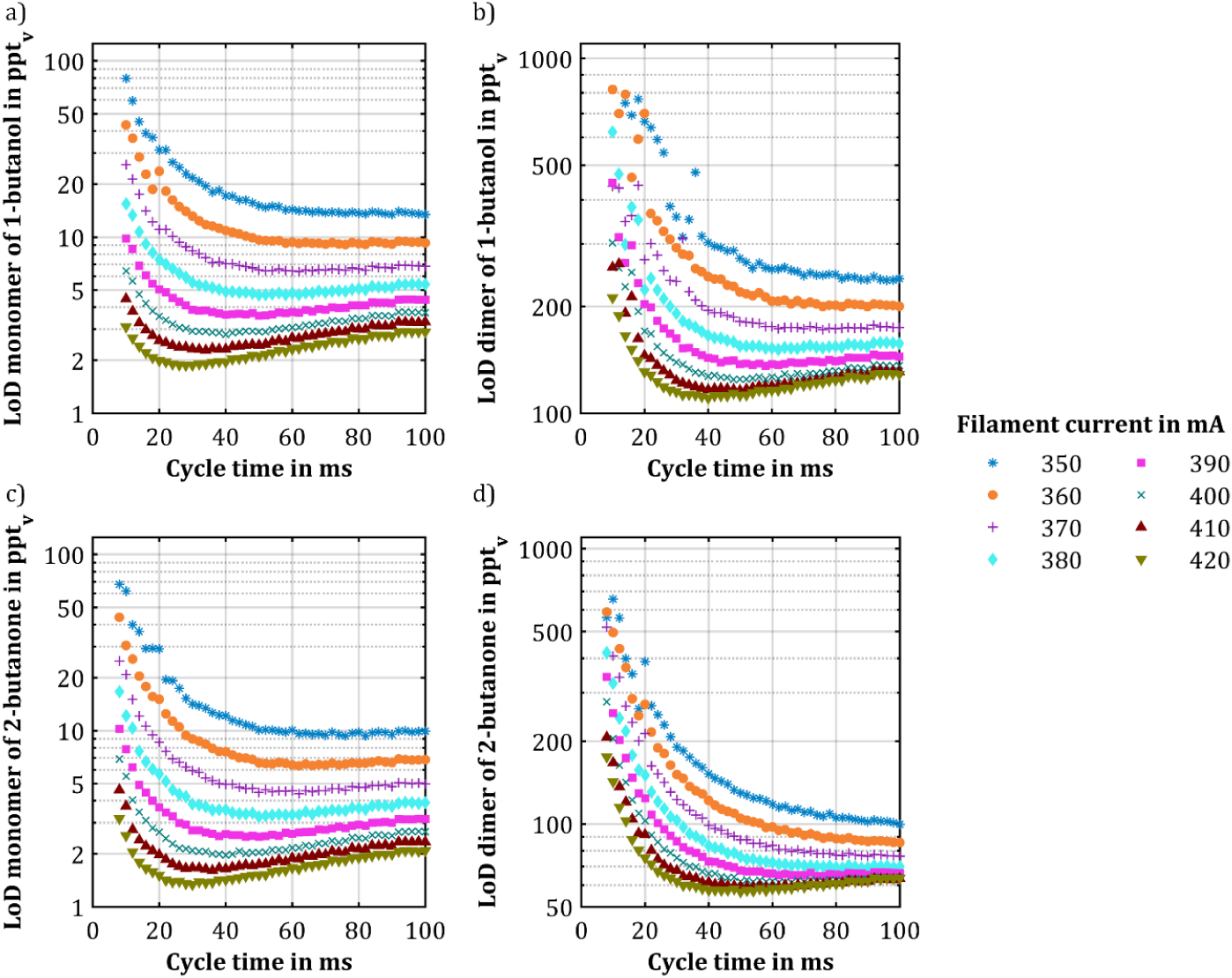
LoD for (a) the protonated monomers and (b) the proton-bound dimers of 1-butanol and (c) the protonated monomers and (d) the proton-bound dimers of 2-butanone plotted over the cycle time at different intensities of the X-ray ionization source, optimal compensation voltage, and an overall measuring time of 1 s (averaging time).

Figure 6 shows the LoDs for the protonated monomers and the proton-bound dimers of 1-butanol and 2-butanone as a function of cycle time for different intensities of the X-ray ionization source. The overall measurement time was limited to 1 s. As expected from the results above, the LoD for the protonated monomers reaches a distinct minimum depending on the intensity of the X-ray ionization source. For even longer cycle times, the LoD increases due to the increased noise as a consequence of less averaging in the given measurement time of 1 s. Of course, the lowest LoD is reached with the highest intensity of the X-ray ionization source. However, with increasing X-ray intensity, the minimum LoD is obtained at shorter cycle times. Using the highest investigated X-ray intensity, the LoD for the protonated monomers of 1-butanol is 1.9 ppt_v_, and for the protonated monomers of 2-butanone it is 1.3 ppt_v_. For the proton-bound dimers, the LoDs are 110 ppt_v_ for 1-butanol and 57 ppt_v_ for 2-butanone.

The observations show critical dependencies of the LoDs on both the cycle time and the intensity of the ionization source, with different optimal conditions for protonated monomers and proton-bound dimers. This emphasizes the need to adapt the cycle time to target the highest signal-to-noise ratio in dependence of the application that defines the maximum measuring time, which leads to the lowest LoD. Since the formation of product ions from reactant ions by APCI strongly depends on the ion mobility, proton affinity, and reaction rate of analytes, and the optimum cycle time for the lowest detection limit is an analyte-specific parameter that cannot be given as one general value. However, as it turned out for the tested alcohol and ketone, the optimum cycle times hardly differ when comparing different monomers with optimum cycle times around 30 ms. The same statement applies to the different dimers of 1-butanol and 2-butanone with optimum cycle times around 45 ms. For better visualization, in Figure S11, the relative detection limits of the protonated monomers and the relative detection limits of the proton-bound dimers of 1-butanol and 2-butanone are plotted against the cycle time. For the setup investigated, it is suggested that a cycle time of around 40 ms presents a good compromise between the lowest LoD for both the protonated monomers and the proton-bound dimers.

## Conclusion

This study aimed to investigate the influence of the ionization source intensity and cycle time of IMS equipped with field-switching ion shutters on the detection limits. The results clearly indicate that higher intensities of the ionization source allow for faster cycle times and improved LoD. It was further observed that the formation of the protonated monomers is slower than the formation of the positive reactant ions, while the formation of the proton-bound dimers is even slower compared to the protonated monomers. This leads to the fact that individual optimal cycle times exist for the protonated monomers and the proton-bound dimers. However, the general trend seems to be consistent across different substances, and a cycle time of around 40 ms was identified as a good compromise, balancing the LoD for both the protonated monomers and proton-bound dimers. It is important to note that, to a certain extent, an elongated cycle time leads to an increased signal-to-noise ratio even though less averaging is possible in a given amount of time. Overall, this work provides valuable insights into how the cycle time of IMS with field-switching ion shutters needs to be set for the highest signal-to-noise ratio, resulting in the lowest detection limits, and how the application affects this setting by possibly limiting the overall measuring time.

## Data Availability

Data will be made available on request.

## Supporting Information Available

Schematic overview of experimental setup, additional simulation, and measurement results as referenced in the manuscript (PDF)
