# Supplementary material for: How Repetition Rate Impacts Detection Limits of Ion Mobility Spectrometers with Field-Switching Ion Shutters
Source: Anal Chem. 2025 Apr 10;97(15):8609–16. doi: 10.1021/acs.analchem.5c01027 (PMC12019778; doi:10.1021/acs.analchem.5c01027)
Supplement: Supplementary file 1 — ac5c01027_si_001.pdf [file ac5c01027_si_001.pdf]

Supporting Information

How repetition rate impacts detection limits of ion mobility spectrometers with field-switching ion shutters

Martin Lippmann*, Moritz Hitzemann, Alexander Nitschke, Stefan Zimmermann

lippmann@geml.uni-hannover.de

Leibniz Universität Hannover, Institute of Electrical Engineering and Measurement Technology, Department of Sensors and Measurement Technology, Appelstr. 9A, 30167 Hannover, Germany

Table of Content

Figure S1: Schematic overview of the IMS equipped with an extended field-switching shutter. The pusher electrode on the left and both neighboring grids are pulsed for operation, while the third grid is integrated into the resistive drift voltage divider to stabilize the electric field within the drift tube. For ionization, an orthogonally mounted X-ray source is used. S2
Figure S2: Simulated RMS values of the residual electric field in the ionization region of different field-switching (FS) shutters in dependence on the compensation voltage. (a) showing the results for a common FS shutter for different optical transparencies of the injection grid, (b) showing the result for an extended FS shutter using grids with 80% optical transparency as introduced by Kirk et al.[1], (c) showing the results for the FS shutter used in this work using grids with 80% optical transparency...S2
Figure S3: RIP+ amplitude over the compensation voltage compared to the RMS value of the residual electric field for different compensation voltages obtained from a COMSOL simulation....S3
Figure S4: Exemplary IMS spectrum with Gaussian fit of the RIP^+ as well as peak position and fit error determined from the fit....S3
Figure S5: Relative amplitude of clean, dry air as sample gas plotted over the cycle time and reaction time, respectively, at different compensation voltages and a constant filament current of 400 mA...S4
Figure S6: Characteristics of the X-ray ionization source, showing the dependence between filament current and anode current (a), the linear relationship between anode current and detector current, when having a constantly opened ion shutter (b) and the square root dependence of charge of the RIP from the anode current (c). S4
Figure S7: Total charge of the RIP^+, the protonated monomer of 1-butanol and the proton-bound dimer of 1-butanol for a concentration of (a) 0 ppt_v, (b) 43 ppt_v and (c) 743 ppt_v 1-butanol in the sample gas. S5
Figure S8: Relative amplitudes for (a) the RIP^+ and (b) the protonated monomer of 1-butanol over the cycle time at optimal compensation voltage but different ionization source intensities, and 48 ppt_v 1-butanol in the sample gas. The intensity of the ionization source was varied by adjusting the filament current of the X-ray source....S5
Figure S9: (a)Measured standard deviation of the zero signal of the IMS over number of averages and square root fit of noise and (b) exemplary representation of standard deviation of the zero signal over the cycle time for a measurement time of 1 s S6
Figure S10: Exemplary signal-to-noise ratio of the protonated monomer of 1-butanol dependent on the cycle time for a measurement time of 1 s and a 1-butanol concentration of 48 ppt_v for different filament currents of the ionization source. S6
Figure S11: Relative LoD for (a) the protonated monomers and (b) the proton-bound dimers of 1-butanol, and (c) the protonated monomers and (d) the proton-bound dimers of 2-butanone plotted over the cycle time at different intensities of the X-ray ionization source, optimal compensation voltage and an overall measuring time of 1 s (averaging time). S7

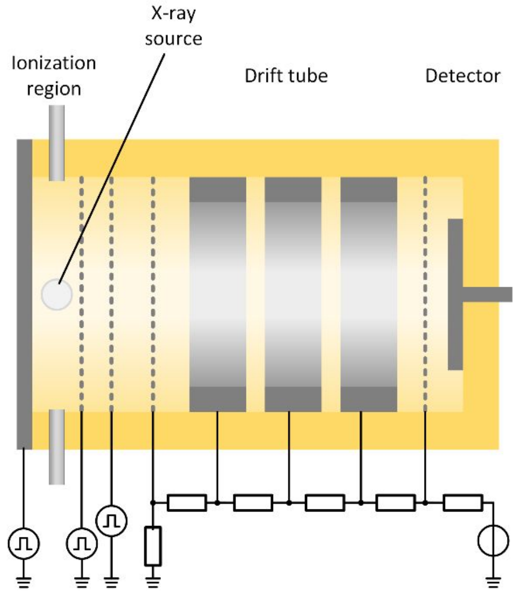

Figure S1: Schematic overview of the IMS equipped with an extended field-switching shutter. The pusher electrode on the left and both neighboring grids are pulsed for operation, while the third grid is integrated into the resistive drift voltage divider to stabilize the electric field within the drift tube. For ionization, an orthogonally mounted X-ray source is used.

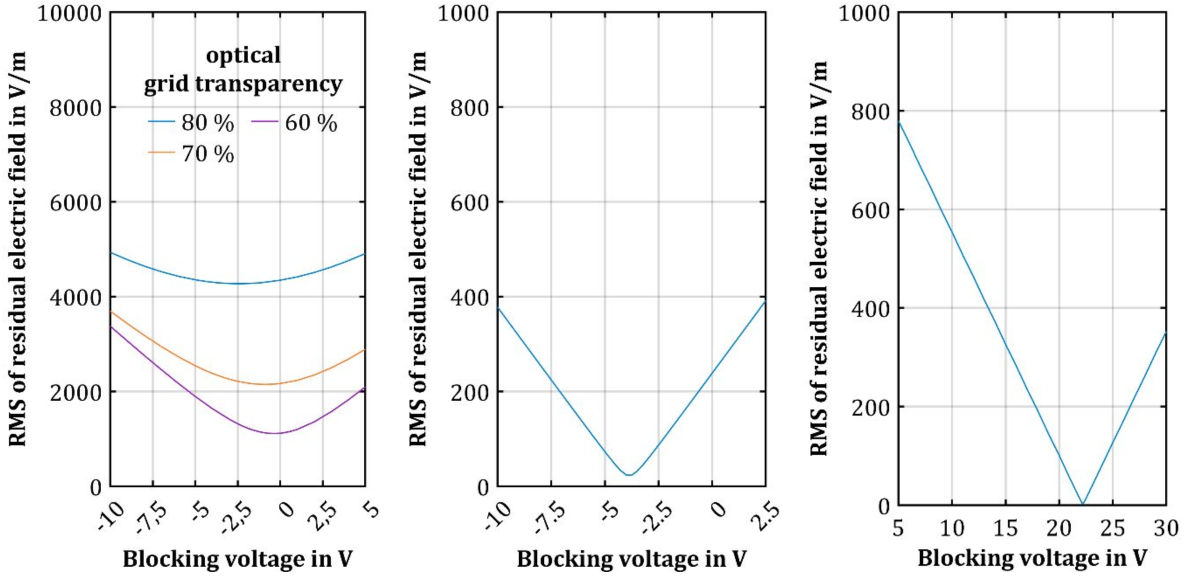

Figure S2: Simulated RMS values of the residual electric field in the ionization region of different field-switching (FS) shutters in dependence on the compensation voltage. (a) showing the results for a common FS shutter for different optical transparencies of the injection grid, (b) showing the result for an extended FS shutter using grids with 80% optical transparency as introduced by Kirk et al.[1], (c) showing the results for the FS shutter used in this work using grids with 80% optical transparency.

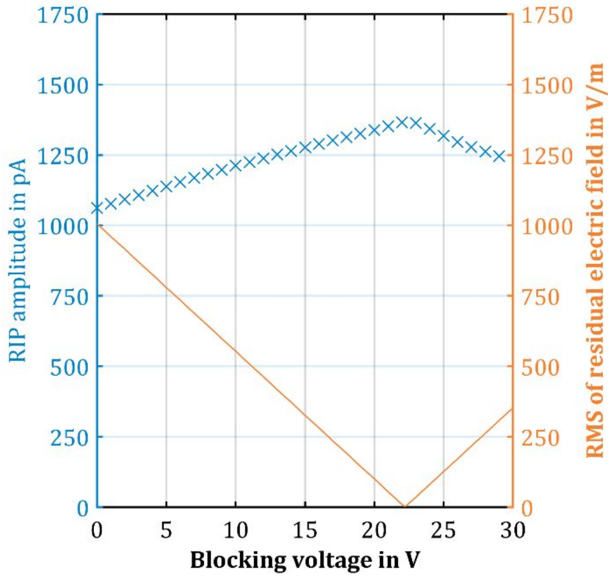

Figure S3: RIP⁺ amplitude over the compensation voltage compared to the RMS value of the residual electric field for different compensation voltages obtained from a COMSOL simulation.

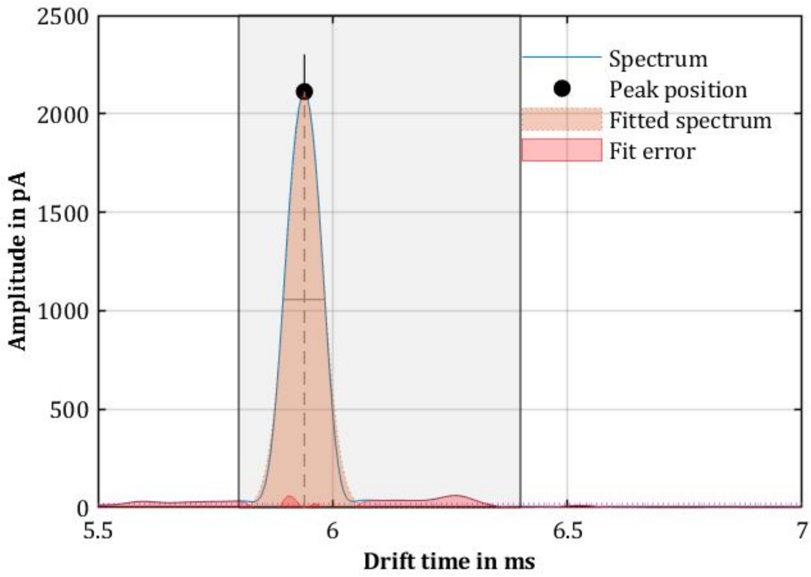

Figure S4: Exemplary IMS spectrum with Gaussian fit of the RIP⁺ as well as peak position and fit error determined from the fit.

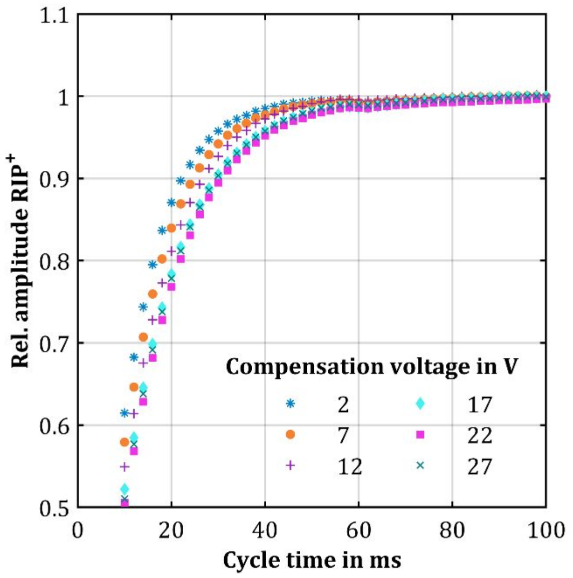

Figure S5: Relative amplitude of clean, dry air as sample gas plotted over the cycle time and reaction time, respectively, at different compensation voltages and a constant filament current of 400 mA.

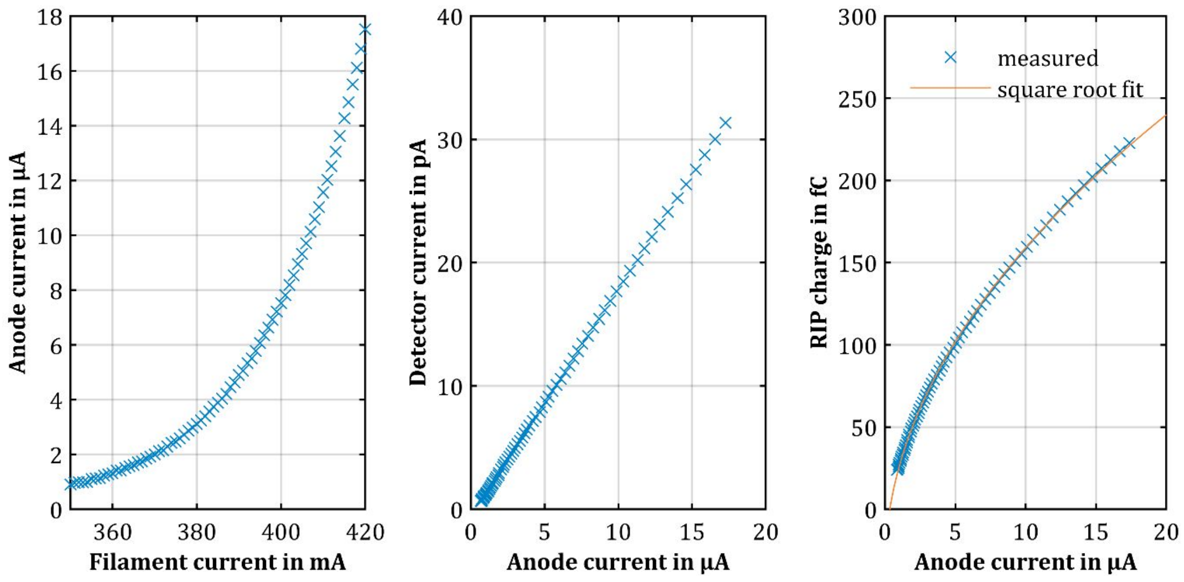

Figure S6: Characteristics of the X-ray ionization source, showing the dependence between filament current and anode current (a), the linear relationship between anode current and detector current, when having a constantly opened ion shutter (b) and the square root dependence of charge of the RIP from the anode current (c).

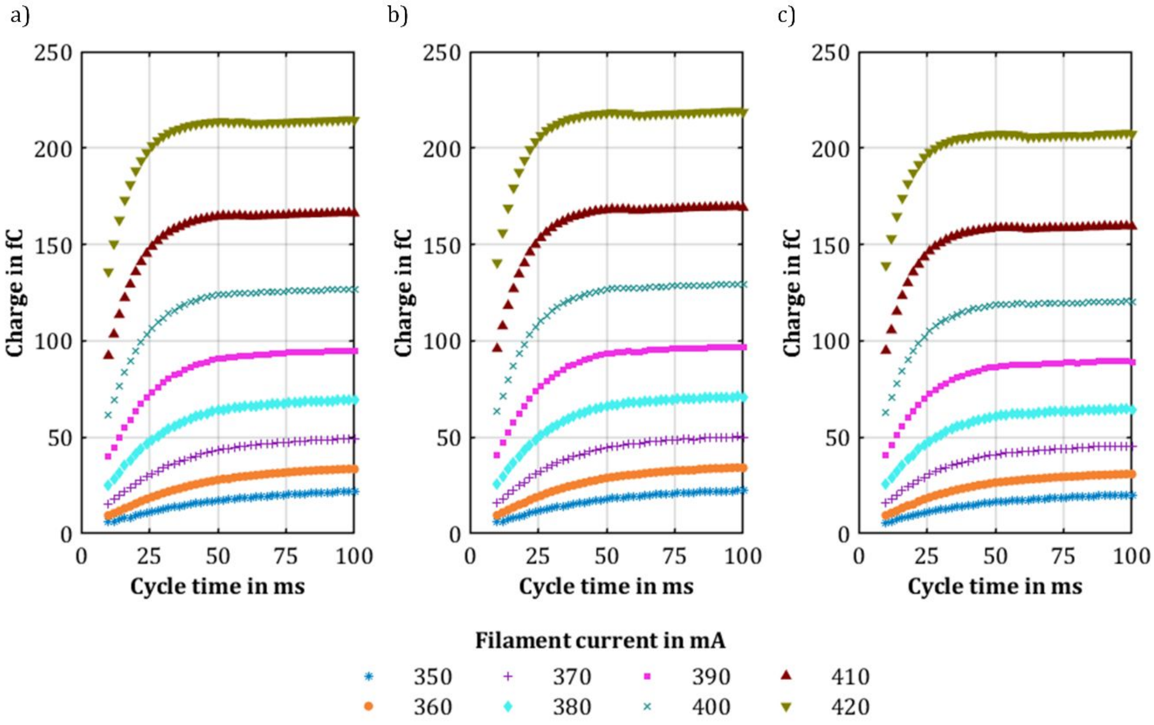

Figure S7: Total charge of the RIP^+, the protonated monomer of 1-butanol and the proton-bound dimer of 1-butanol for a concentration of (a) 0 ppt_v, (b) 43 ppt_v and (c) 743 ppt_v 1-butanol in the sample gas.

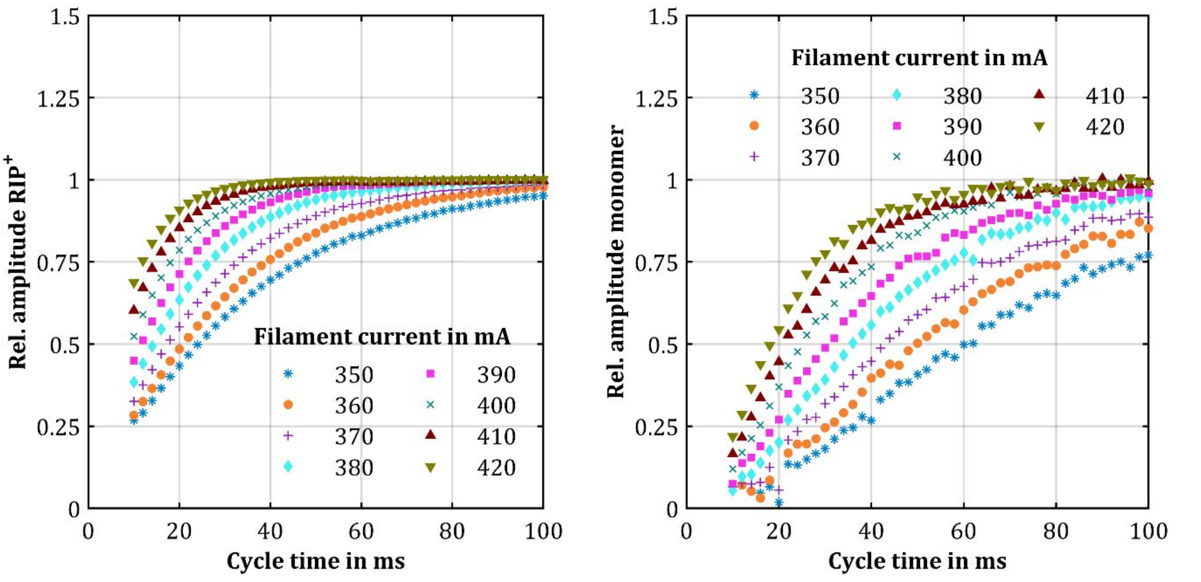

Figure S8: Relative amplitudes for (a) the RIP^+ and (b) the protonated monomer of 1-butanol over the cycle time at optimal compensation voltage but different ionization source intensities, and 48 ppt_v 1-butanol in the sample gas. The intensity of the ionization source was varied by adjusting the filament current of the X-ray source.

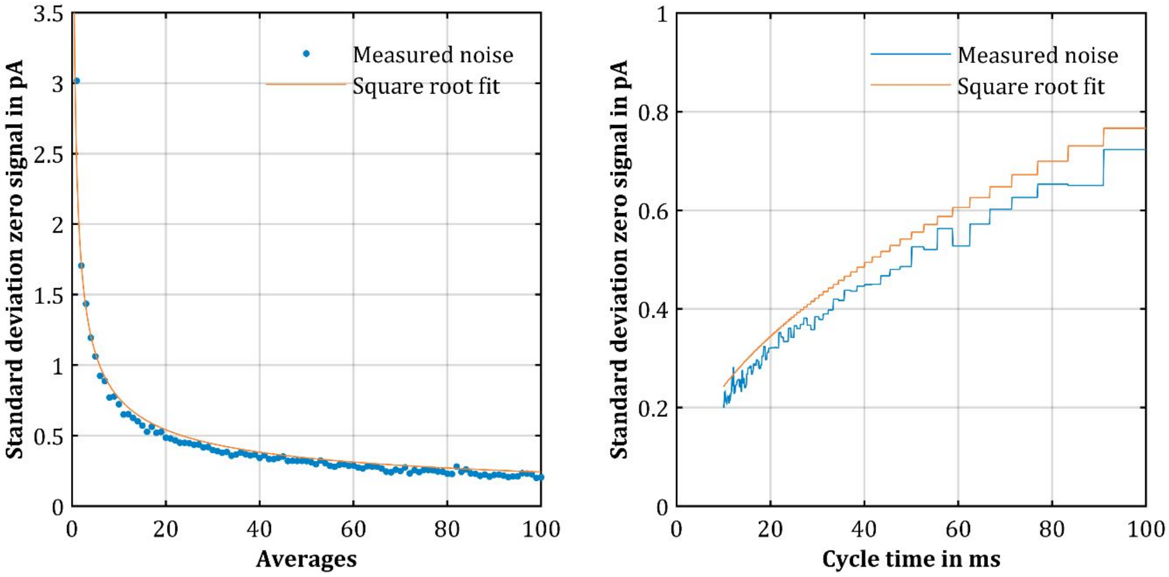

Figure S9: (a) Measured standard deviation of the zero signal of the IMS over number of averages and square root fit of noise and (b) exemplary representation of standard deviation of the zero signal over the cycle time for a constant total measurement time of 1 s.

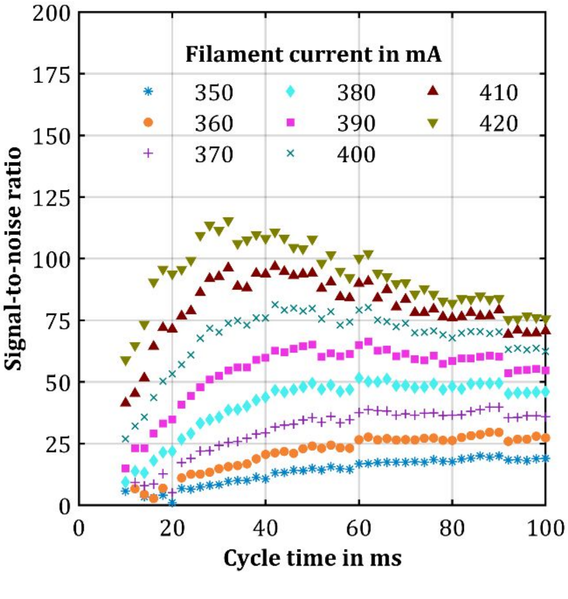

Figure S10: Exemplary signal-to-noise ratio of the protonated monomer of 1-butanol dependent on the cycle time for a measurement time of 1 s and a 1-butanol concentration of 48 ppt$_v$ for different filament currents of the ionization source.

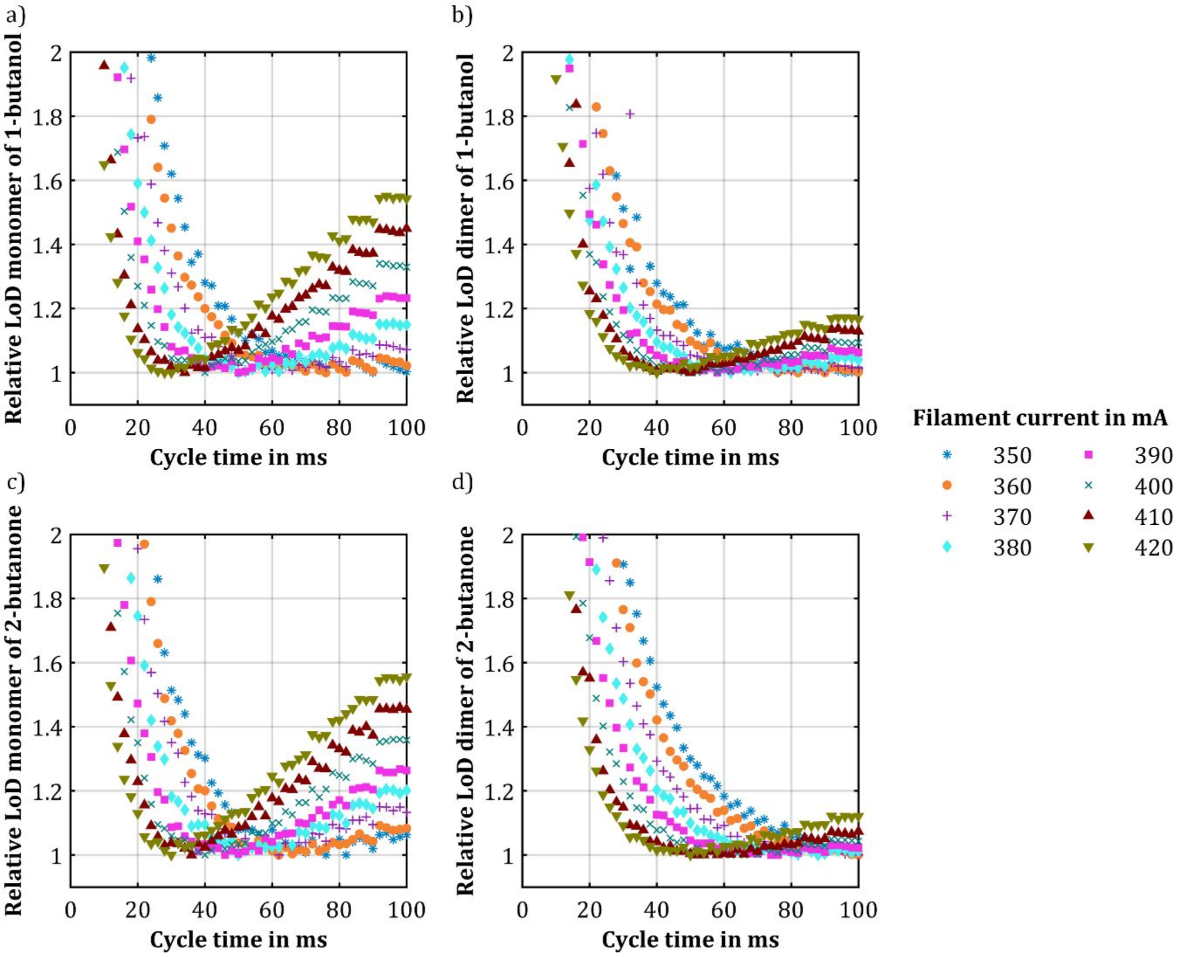

Figure S11: Relative LoD for (a) the protonated monomers and (b) the proton-bound dimers of 1-butanol, and (c) the protonated monomers and (d) the proton-bound dimers of 2-butanone plotted over the cycle time at different intensities of the X-ray ionization source, optimal compensation voltage and an overall measuring time of 1 s (averaging time).

References

(1) Kirk, A. T.; Küddelsmann, M. J.; Bohnhorst, A.; Lippmann, M.; Zimmermann, S. Improving Ion Mobility Spectrometer Sensitivity through the Extended Field Switching Ion Shutter. *Anal. Chem.* **2020**, *92*, 4838-4847.
